# Coexistence of papulonecrotic tuberculid and Poncet’s disease: A case report of dual tuberculin hypersensitivity reactions and literature review

**DOI:** 10.1097/MD.0000000000049546

**Published:** 2026-06-26

**Authors:** Zhubiao Ye, Li-Tian Ma, Su Wang, Shuna Zhang, Yujian Ye

**Affiliations:** a Department of Dermatology, Hangzhou Third People’s Hospital, Hangzhou, Zhejiang, China; b Department of Thoracic Surgery, Tangdu Hospital, Fourth Military Medical University (Air Force Medical University), Xi’an, China; c Department of Traditional Chinese Medicine, Tangdu Hospital, Fourth Military Medical University (Air Force Medical University), Xi’an, China; d Shaanxi Provincial Key Laboratory of Integrated Traditional Chinese and Western Medicine in Oncology Diagnosis and Treatment, Xi’an, China; e School of Medicine, Northwest University, Xi’an, China; f Department of Dermatology, Haining Maternity and Child Health Care Hospital, Haining, Zhejiang, China.

**Keywords:** case report, papulonecrotic tuberculid, Poncet’s disease, tuberculosis hypersensitivity

## Abstract

**Rationale:**

Papulonecrotic tuberculid (PNT) and Poncet’s disease (PD) are rare, immune-mediated reactions to *Mycobacterium tuberculosis* (MTB) that occur without direct bacterial isolation. The concurrent manifestation of both conditions in a single individual is exceptionally rare, with only limited cases documented in the literature. This report documents a singular case of concurrent PNT and PD, illustrating multisystem delayed-type hypersensitivity as an exceptional manifestation of MTB.

**Patient concerns:**

A 30-year-old male presented with a 3-year history of recurrent, symmetrical necrotic papules on the extremities and trunk, evolving through sequential stages from papule to central necrosis, crusting, and varioliform scarring. He also reported episodes of inflammatory polyarthritis involving the knees, elbows, wrists, and metacarpophalangeal joints. He had sustained household contact with a grandmother diagnosed with pulmonary tuberculosis.

**Diagnoses:**

Laboratory testing revealed elevated total immunoglobulin E (>2000 IU/mL), an increased erythrocyte sedimentation rate (ESR = 31 mm/h), and elevated C-reactive protein (CRP = 29 mg/L). Tuberculin skin testing was positive (13 mm × 13 mm), and interferon-gamma release assay was positive. Skin biopsy revealed leukocytoclastic vasculitis, epithelioid necrotizing granulomas, and multinucleated giant cells. He was diagnosed with PNT and PD as hypersensitivity reactions to latent MTB infection.

**Interventions:**

Standard antitubercular therapy (isoniazid, rifampicin, and ethambutol) was administered.

**Outcomes:**

Complete resolution of cutaneous and articular symptoms was achieved within the 6-month follow-up period.

**Lessons:**

Concurrent PNT and PD underscore the potential of MTB to trigger synchronous multisystem immune reactions. The observed elevated immunoglobulin E levels suggest a possible T helper 2-skewed immunologic background, broadening the understanding of tuberculous hypersensitivity syndromes. In tuberculosis-endemic areas, clinicians should maintain a high index of suspicion for such atypical presentations to avoid diagnostic delays and prevent chronicity. Anti-MTB therapy remains the definitive and effective treatment for these immune-mediated phenotypes.

## 1. Introduction

Tuberculosis (TB), an enduring global health burden caused by *Mycobacterium tuberculosis*, is increasingly recognized not merely as an infectious disease but also as a significant instigator of diverse immune-mediated phenomena. The host response to mycobacterial antigens can transcend the local containment of the bacillus, giving rise to sterile, hypersensitivity-driven syndromes that affect organs distant from the primary site of infection.^[1,2]^ Within this spectrum, papulonecrotic tuberculid (PNT) and Poncet’s disease (PD) represent 2 distinct yet pathophysiologically linked clinical entities. PNT is an uncommon cutaneous tuberculid characterized by recurrent, symmetric crops of dusky red papules that evolve to undergo central necrosis and crusting, typically located on the extensor surfaces of the limbs and healing with distinctive atrophic, varioliform scars.^[3]^ Histopathological examination is crucial, revealing leukocytoclastic or lymphocytic vasculitis accompanied by fibrinoid necrosis and wedge-shaped dermal infarction, all in the notable absence of demonstrable acid-fast bacilli within the lesions.^[4]^ Conversely, PD, also termed tuberculous rheumatism, is defined as a reactive, aseptic polyarthritis associated with an active extra-articular tuberculous focus. It typically manifests as an acute, often migratory, oligo- or polyarthritis predominantly affecting large joints such as the knees and ankles.^[5,6]^ A pivotal diagnostic feature is the sterile inflammatory nature of the synovial fluid, and the diagnosis is heavily contingent upon excluding other causes of inflammatory arthritis while observing a dramatic and prompt clinical response to the initiation of antitubercular therapy (ATT).^[7]^

Beneath these clinical phenotypes lies a complex network of immunological dysregulation that orchestrates the tissue-destructive manifestations of tuberculids. The pathogenesis of both PNT and PD is rooted in a dysregulated adaptive immune response to mycobacterial antigens, wherein the interplay between innate and adaptive immune effectors determines disease expression. Emerging evidence from related granulomatous diseases underscores the pivotal role of immune checkpoint pathways in modulating T-cell reactivity against mycobacterial infections. In lepromatous leprosy – a chronic mycobacterial disease caused by *Mycobacterium leprae* – programmed death-1 (PD-1) and its ligands are differentially expressed across T cells, B cells, regulatory T cells, and monocytes, with PD-1 overexpression correlating with disease severity, bacteriological index, and T-cell anergy. Notably, in vitro blockade of PD-1 restores regulatory T-cell suppressive function and enhances interleukin-10 (IL-10) secretion, suggesting that analogous checkpoint dysregulation may contribute to the systemic hypersensitivity observed in tuberculids.^[8]^ Concurrently, CD40-mediated co-stimulatory signaling represents a critical hub in antigen-specific T-cell activation and effector differentiation. Khan and colleagues demonstrated that the protein tyrosine phosphatase SHP-1 plays a crucial reciprocal role in regulating CD40 signaling in immune cells, thereby fine-tuning the threshold of T-cell activation and potentially influencing the intensity of delayed-type hypersensitivity responses to mycobacterial antigens.^[9]^

At the effector level, the innate immune arm contributes substantially to the sterile inflammatory phenotype characteristic of tuberculids. The NOD-, LRR-, and pyrin domain-containing protein 3 (NLRP3) inflammasome serves as a central platform translating persistent mycobacterial antigen recognition into tissue-destructive inflammatory cascades in the absence of viable bacilli. Recent investigations have identified interleukin-11 (IL-11) as a potent inducer of NLRP3 inflammasome activation in monocytes, driving nuclear factor-κB (NF-κB) signaling, interleukin-1β (IL-1β) maturation, and promoting inflammatory cell migration to target tissues – mechanisms that may underlie the cutaneous necrosis and synovial inflammation observed in PNT and PD, respectively.^[10]^ Complementing this innate axis, IL-11 has also been shown to promote the differentiation of pathogenic T helper 17 (Th17) cells that produce interleukin-17A (IL-17A), granulocyte-macrophage colony-stimulating factor (GM-CSF), and interleukin-21 (IL-21), expanding the repertoire of T-cell-mediated tissue injury beyond classical T helper 1 (Th1) responses and implicating Th17-driven neutrophilic inflammation in the vascular damage seen in papulonecrotic lesions.^[11]^ Furthermore, the metabolic and epigenetic landscape of immune cells profoundly shapes their functional commitment during chronic antigenic stimulation. Sirtuins, a family of NAD+-dependent deacetylases, have emerged as key metabolic regulators that influence inflammatory responses, cellular stress adaptation, and immune cell fate decisions across diverse pathological contexts, including pancreatic and prostate malignancies.^[12,13]^ Their demonstrated ability to modulate nuclear factor-κB signaling, glycolytic metabolism, and androgen receptor pathways suggests potential, albeit currently unexplored, roles in controlling the immunometabolic programming of T cells and macrophages during chronic tuberculous hypersensitivity reactions. Finally, the broader paradigm of immune-mediated tissue injury converges on shared principles of dysregulated immune activation across seemingly disparate disease contexts. In hematological malignancies such as diffuse large B-cell lymphoma, the tumor microenvironment is shaped by complex interactions between malignant cells and infiltrating immune effectors, wherein checkpoint signaling, metabolic adaptation, and antigen-specific targeting determine disease outcomes.^[14]^ Engineered immune cell therapies, including chimeric antigen receptor T-cell strategies predicated on the precise identification of disease-specific target antigens, exemplify how directed immune responses can be harnessed therapeutically.^[15]^ Collectively, these insights underscore that dysregulated immune activation – whether directed against tumor antigens, autologous tissues, or mycobacterial epitopes – frequently converges on common innate and adaptive effector pathways that mediate tissue destruction.

Although both PNT and PD are understood to represent delayed-type hypersensitivity reactions to mycobacterial antigens, they conventionally present as isolated phenomena involving the skin and joints, respectively. The concurrent manifestation of both conditions in a single individual is an exceptionally rare clinical event, with only scarce cases documented in the medical literature.^[16-18]^ This rarity, combined with their nonspecific and overlapping presentations, creates a substantial diagnostic challenge. Patients presenting with concomitant skin and joint symptoms are frequently misdiagnosed with more common conditions such as psoriasis with psoriatic arthritis, systemic vasculitis, or other connective tissue diseases, leading to critical delays in appropriate treatment and potential harm from misguided immunosuppressive therapy.^[19,20]^

The cornerstone of management for both PNT and PD is the eradication of the underlying TB infection with a standard ATT regimen. Both conditions characteristically exhibit a prompt and favorable resolution of symptoms upon initiation of appropriate antimicrobial therapy.^[21,22]^ Consequently, early and accurate recognition is paramount to avoid the unnecessary and potentially detrimental use of corticosteroids or disease-modifying antirheumatic drugs.^[5]^ Despite their shared etiological trigger and therapeutic response, the precise immunopathological pathways that link these cutaneous and articular manifestations remain incompletely elucidated, and specific biomarkers for this systemic hypersensitivity state are currently lacking.^[23]^ This report aims to detail the clinical presentation of this rare dual manifestation, providing a structured diagnostic framework to aid clinicians in its identification. Furthermore, it seeks to contribute clinical evidence toward a more integrated understanding of TB as a cause of multisystem, immune-mediated disease, underscoring the necessity of a high index of suspicion in appropriate epidemiological contexts.

## 2. Patient information

We herein report the case of a 30-year-old male who presented to our dermatology clinic with a history of recrudescent, symmetrical erythematous papules distributed across his trunk and limbs. The patient first developed these cutaneous manifestations approximately 3 years prior to his presentation. At initial onset in February 2019, lesions appeared as erythematous papules that characteristically evolved through distinct, sequential morphological stages: each lesion progressed to form central pustules and necrosis, subsequently crusted over, and ultimately resolved with distinctive atrophic, varioliform scarring. This stepwise progression from papule to scar – repeatedly observed across multiple lesion cycles over the disease course – constitutes the classic pattern pathognomonic for papulonecrotic tuberculid. This cyclical pattern, marked by the continuous emergence of new lesions as older ones healed, was a hallmark of his disease course. Concurrent with the cutaneous manifestations, the patient also reported episodes of pain and stiffness in multiple joints, suggesting possible systemic involvement. During the 3-year interval following initial symptom onset, the patient was misdiagnosed with psoriatic arthritis and treated with methotrexate (MTX) 10 mg weekly and topical corticosteroids, resulting in temporary symptom resolution. Despite ongoing immunosuppressive therapy, new lesions continued to emerge and followed the same characteristic stepwise evolution – progressing from papule to pustule, then to central necrosis and crusting, and finally healing with pitted scarring. One month prior to his current presentation in March 2022, the patient experienced a severe recurrence in which lesions again manifested as erythematous pustular papules with prominent central necrosis and crusting, accompanied by arthralgia affecting the knees, elbows, wrists, and metacarpophalangeal joints. Acitretin 30 mg daily was prescribed for 2 weeks but did not alleviate his symptoms. Upon retrospective inquiry, the patient ruled out recent travel to tuberculosis-endemic areas and affirmed no occupational hazards such as employment in healthcare, mining, or congregate settings. Nevertheless, the patient reported sustained household contact with his grandmother during the period preceding symptom onset. His grandmother had reportedly received a clinical diagnosis of pulmonary tuberculosis in the past, based on her presentation with persistent fever, nocturnal diaphoresis, and hemoptysis; however, this epidemiological history was obtained retrospectively from the patient and could not be independently verified by medical records or microbiological documentation from his grandmother. Despite this inherent limitation, it is well established that prolonged household exposure to an individual with symptomatic, untreated pulmonary tuberculosis constitutes one of the strongest recognized risk factors for latent *M tuberculosis* infection and subsequent tuberculid development.

The publication of this case report was approved by the Ethics Committee of Hangzhou Third People’s Hospital (approval number: 2025KA294). Written informed consent was obtained from the patient for publication of this case report and accompanying images.

## 3. Clinical findings

**Figure 1.**
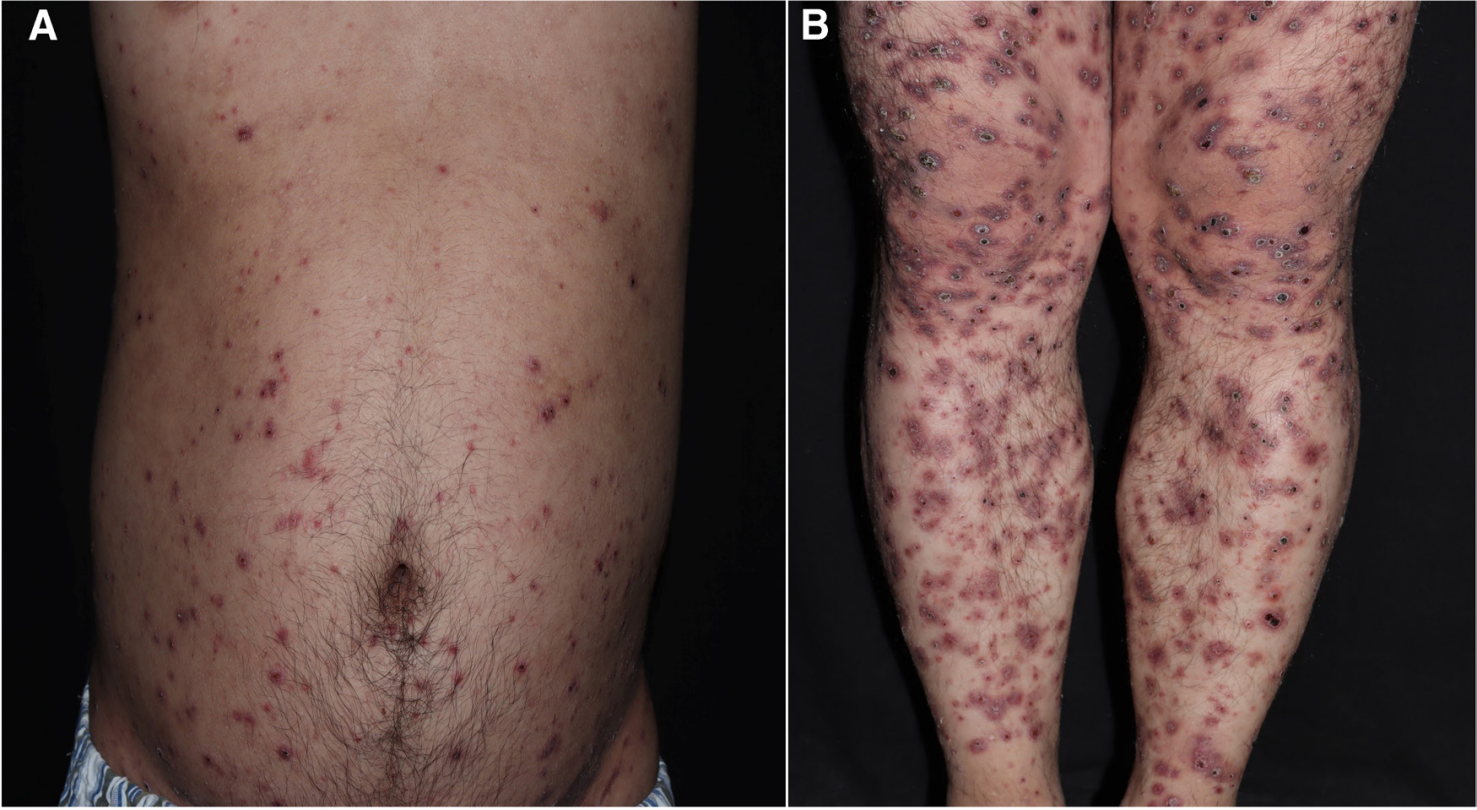
Clinical presentation of papulonecrotic tuberculid (PNT) in a 30-year-old male. (A) Truncal involvement showing symmetrical erythematous papulonodular lesions with central necrosis, crusting, and varioliform (pitted) scarring, indicative of the chronic, recurrent nature of PNT. (B) Limb distribution featuring similar papulopustular and necrotizing lesions, alongside healed atrophic scars, highlighting the characteristic symmetry and multifocal presentation of cutaneous tuberculosis hypersensitivity. PNT = papulonecrotic tuberculid.

Given the lack of a definitive diagnosis for his recurrent rash and arthralgia, the patient was admitted for inpatient care. Dermatological examination revealed widespread erythematous papules, crusts, and atrophic scars, symmetrically involving the trunk and extremities (Fig. 1). There was tenderness to palpation in the elbows, wrists, metacarpophalangeal joints, and knees bilaterally. No significant swelling or deformity was observed in these joints.

## 4. Timeline

**Table:** 

Event	Date
Symptom onset	February 2019
Symptom worsen	February 15, 2022
Hospital admission	March 28, 2022
Laboratory testing	March 29, 2022
Skin biopsy	March 30, 2022
Initiation of isoniazid, rifampicin, ethambutol and celecoxib	April 8, 2022
Discharged from hospital	April 12, 2022
Follow-up at 6 months	October 12, 2022

## 5. Diagnostic assessment

**Figure 2.**
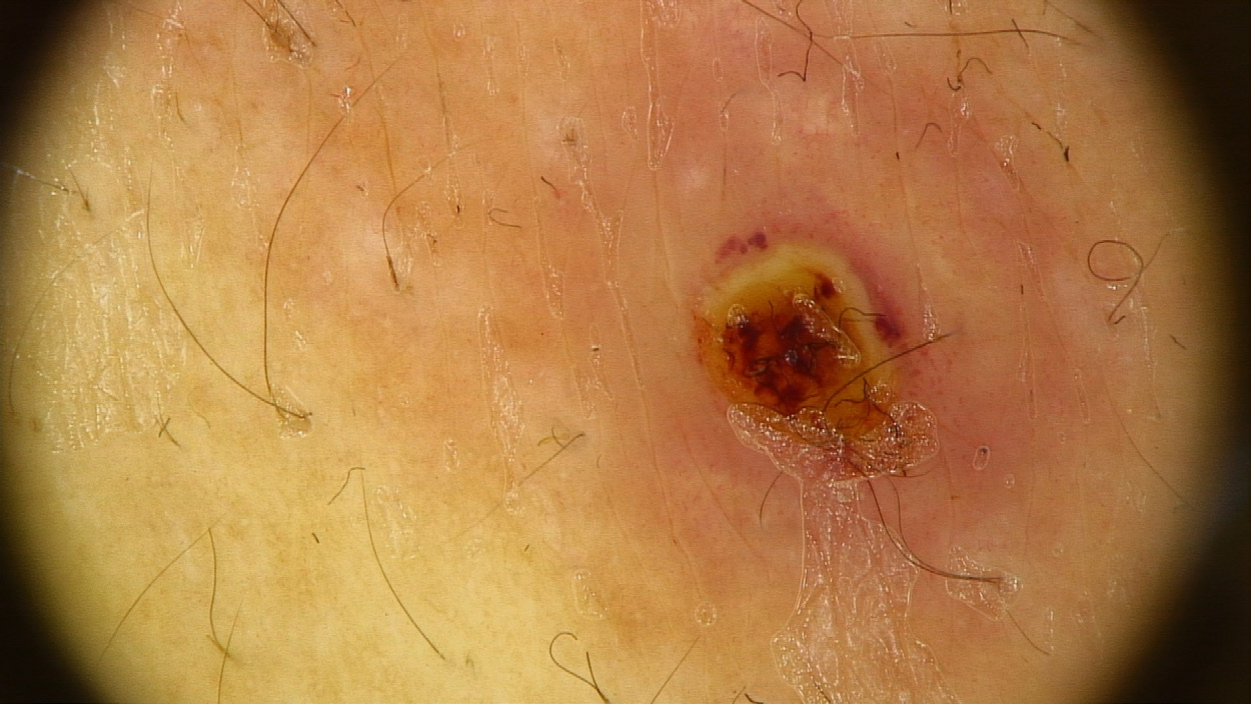
Dermatological imaging finding. Dermoscopy of a papulonecrotic lesion shows a central pustule with partial crusting and an erythematous halo. Punctate and linear vessels are visible, without a consistent pattern of glomerular or circular vessels.

**Figure 3.**
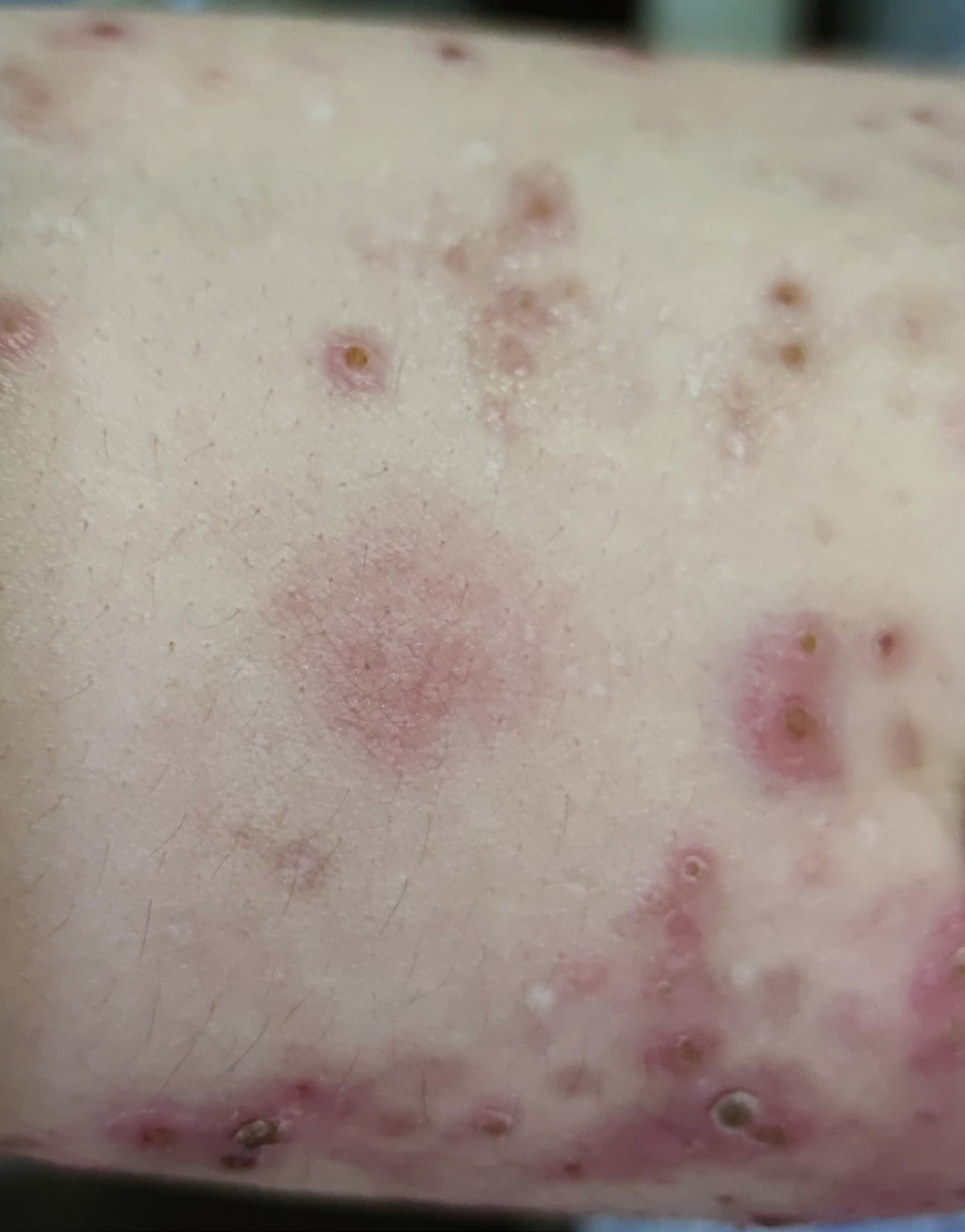
Strongly positive tuberculin skin test (Mantoux reaction). The image shows an induration measuring 13 mm × 13 mm at 72 hours, confirming delayed-type hypersensitivity to *Mycobacterium tuberculosis* antigens. This result supported the immune-mediated pathogenesis of both PNT and Poncet’s disease in the absence of active bacterial isolation. PNT = papulonecrotic tuberculid.

**Figure 4.**
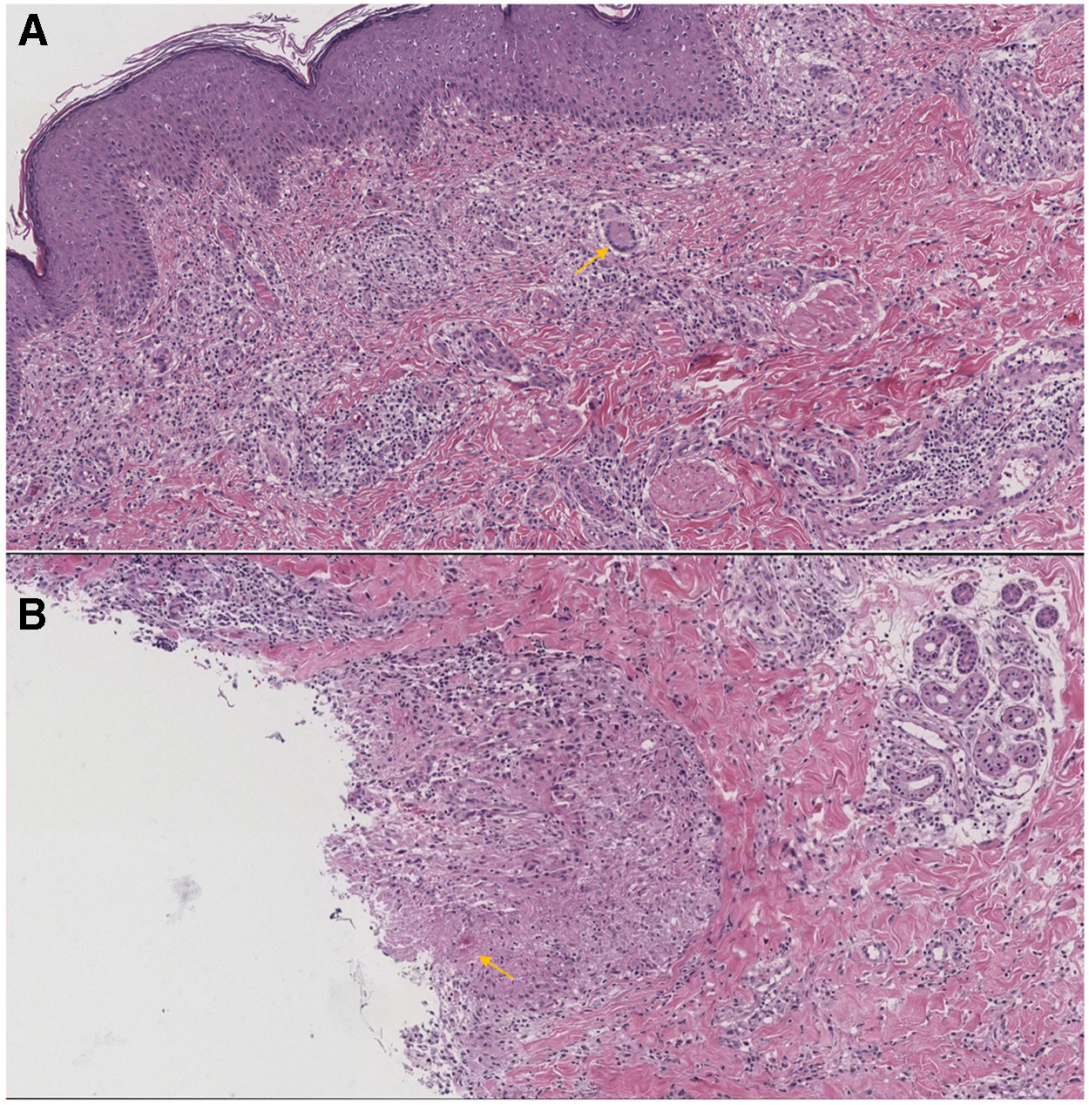
Characteristic histopathological findings. (A) Histopathological examination (hematoxylin and eosin staining, ×100 magnification) of a skin lesion reveals a dense dermal inflammatory infiltrate composed of lymphocytes, histiocytes, and multinucleated giant cells (arrow), consistent with a granulomatous reaction. (B) Pathological examination of tissues (×100 magnification) demonstrates a well-formed epithelioid cell granuloma in the mid-dermis with central caseous necrosis (arrow), surrounded by a lymphocytic cuff.

Routine laboratory workup was unremarkable except for elevated total immunoglobulin E levels (total IgE > 2000 IU/mL), an increased erythrocyte sedimentation rate (ESR = 31 mm/h), and increased C-reactive protein levels (CRP = 29 mg/L). Dermoscopy suggests a diagnosis of papulonecrotic tuberculid rather than psoriasis (Fig. 2). Tuberculin testing was positive (13 mm × 13 mm) at 72 hours (Fig. 3), and interferon-gamma release assays (IGRA) indicated a positive T-spot TB assay. A chest computed tomography (CT) scan showed no abnormalities. Magnetic resonance imaging (MRI) of the right hand and knees revealed slight synovial fluid retention without bone erosion or joint destruction. Skin biopsy revealed leukocytoclastic vasculitis in the dermis, together with a localized epithelioid necrotizing granuloma with multinucleated giant cells (Fig. 4). The clinical, laboratory, and pathological findings collectively indicated a diagnosis of PNT and PD.

## 6. Therapeutic intervention

The patient was initially treated with acitretin capsules at a dose of 30 mg once daily. After the skin biopsy confirmed a diagnosis of PNT and PD, the therapeutic regimen was changed to include isoniazid (300 mg once daily), rifampicin (0.45 g once daily), and ethambutol (0.75 g once daily). In addition, intermittent oral administration of celecoxib was initiated for symptomatic relief.

## 7. Follow-up and outcomes

He achieved nearly complete remission of his recurrent rash and joint pain following the completion of combined therapy. No adverse or unanticipated events were observed during the 6-month follow-up period. The patient was scheduled for a follow-up clinical reassessment to monitor for potential recrudescence of disease activity, with instructions for vigilant self-monitoring regarding the recurrence of cutaneous lesions or articular manifestations.

## 8. Discussion

This case report delineates a patient presenting with the cardinal manifestations of papulonecrotic tuberculid (PNT) and Poncet’s disease (PD) occurring concurrently. The diagnosis of PNT was substantiated by the presence of symmetric, necrotic papulonodular lesions localized to the extremities, a strongly positive tuberculin skin test, and histopathological evidence consistent with paucibacillary granulomatous vasculitis. The diagnosis of PD was established based on the acute onset of a nonerosive, symmetric polyarthritis in the context of documented extrapulmonary tuberculosis, with complete resolution of arthritic symptoms following the initiation of ATT. The co-occurrence of these 2 distinct clinical entities is exceptionally rare, with PNT itself accounting for only approximately 4% of all cutaneous TB cases.^[3]^ The simultaneous presentation of PNT and PD in a single patient represents a noteworthy clinical event that provides a unique perspective into the systemic nature and heterogeneity of hypersensitivity reactions driven by mycobacterial antigens.^[5,6]^ The synchronous manifestation of PNT and PD remains an exceptionally rare occurrence in the medical literature, with only a limited number of documented cases explicitly describing this dual presentation.^[16,17]^ This report contributes a definitive example to this scarce body of evidence, reinforcing the concept that MTB infection can trigger synchronous immune-mediated pathology across disparate organ systems.

The clinical presentation of our patient exemplifies a classic illustration of the established diagnostic frameworks for both PNT and PD, while simultaneously presenting subtleties that merit further discourse. The diagnosis of PNT hinges upon a triad of clinical morphology (symmetrical, necrotic papules), evidence of mycobacterial sensitivity (a strongly positive Mantoux test or IGRA), and corroborative histopathology (wedge-shaped dermal necrosis, vasculitis, and granulomatous inflammation) in the absence of detectable bacilli.^[3,4]^ Our case unequivocally satisfied all these components, closely mirroring the diagnostic trajectory outlined in seminal literature on PNT. For PD, the application of structured diagnostic criteria is paramount. The diagnosis was formally established using the criteria proposed by Sharma and Pinto, which define “definite” PD by the fulfillment of 3 major criteria: nonerosive, nondeforming arthritis, evidence of concomitant extra-articular tuberculosis, and complete resolution of arthritic symptoms following the initiation of ATT.^[7,24]^ Our patient met all 3 major criteria, thereby securing a robust, literature-anchored diagnosis, in contrast to instances where the diagnosis remains provisional due to incomplete fulfillment of these pillars. It is noteworthy that the diagnosis of PD in this case rests upon an important nuance. The Sharma-Pinto criteria for “definite” PD require evidence of concomitant active extra-articular tuberculosis. In our patient, while the strongly positive tuberculin skin test and IGRA confirm MTB sensitization, and the characteristic PNT histopathology supports a tuberculous etiology, no active extra-articular tuberculous focus – such as lymphadenitis, urogenital TB, or osteoarticular TB – was identified on imaging or biopsy. PNT itself represents a hypersensitivity reaction rather than an active infectious lesion. Therefore, the fulfillment of the second Sharma-Pinto criterion hinges on the interpretation of PNT (together with positive immunologic assays) as sufficient evidence of underlying tuberculous infection. This interpretation, while clinically justified by the dramatic response to ATT and consistent with established practice in tuberculid diagnosis, acknowledges a degree of diagnostic uncertainty inherent to cases where the primary infectious focus remains occult. A point of potential divergence and diagnostic complexity, alluded to in the initial framework, could involve an initial, partial clinical response to an immunomodulatory agent such as MTX prior to the administration of ATT. A scenario involving a transient response to MTX would introduce a significant diagnostic conundrum. Although ATT is recognized as the definitive curative intervention in classical descriptions, a temporary symptomatic amelioration with MTX would powerfully underscore the predominant immune-mediated pathophysiology of these conditions.^[25]^ It would demonstrate that while suppression of the inflammatory cascade can yield a salutary effect, it fails to address the underlying antigenic source, inevitably culminating in disease recrudescence or incomplete control.^[19]^ This pattern, while potentially misleading, serves to distinguish such cases from straightforward reports of isolated PNT or PD responding exclusively to ATT, and it highlights the diagnostic challenges encountered when immune-mediated symptoms are partially modifiable by nonspecific immunosuppression. Collectively, this case contributes to the growing evidence base for this rare syndromic association, advancing its status from anecdotal observation toward a more delineated clinical phenotype.

The observed elevation in serum IgE levels (total IgE > 2000 IU/mL) in our patient may signify a heightened Th2-skewed immune milieu, which has been previously documented in certain forms of tuberculous hypersensitivity.^[11,26]^ In the context of PNT and PD, mycobacterial antigens can drive a mixed Th1/Th2 response, with the elevated IgE potentially reflecting a broader state of immune dysregulation, including heightened eosinophilic and mast-cell activity, that may amplify tissue inflammation in both cutaneous and articular tissues. This immunologic deviation underscores the systemic nature of the hypersensitivity reaction and aligns with prior reports of hyperglobulinemia in patients presenting with tuberculids.^[27]^

The central enigma posed by this clinical presentation concerns the pathophysiological mechanism enabling the synchronous emergence of PNT and PD within a single host. We hypothesize that this phenomenon likely stems from an unusually robust and systemic delayed-type (Type IV) hypersensitivity reaction to MTB antigens.^[28]^ An active tuberculous focus, potentially an occult subclinical reservoir such as a dormant lymph node or other extrapulmonary site not radiologically apparent, may serve as a persistent antigenic reservoir, continuously shedding bacterial components into the systemic circulation. Although chest CT did not demonstrate overt lymphadenopathy or parenchymal lesions, the strongly positive IGRA and tuberculin skin test confirm prior sensitization to MTB antigens, implying the existence of a subclinical infectious focus capable of driving the systemic hypersensitivity response. These circulating antigens or their resulting immune complexes may then deposit in tissues possessing susceptible microvasculature. Within the dermal vasculature, this deposition incites the leukocytoclastic vasculitis and focal necrosis pathognomonic of PNT. Concurrently, a parallel process within the synovial membrane – potentially amplified by molecular mimicry between mycobacterial heat-shock proteins and human cartilage antigens, a theory under investigation in PD pathogenesis – precipitates the characteristic sterile inflammation of the joint.^[29,30]^

This hypothesized sequence of events finds indirect corroboration in the patient’s clinical trajectory. An initial, albeit partial, response to MTX, an agent that modulates T-cell proliferation and function, directly implicates a cell-mediated immune pathway in driving disease activity. However, the sustained and complete resolution of symptoms only upon initiation of ATT conclusively identifies the persistent MTB antigens as the fundamental driver of this systemic immune dysregulation. This clinical progression – from a transient response to broad immunosuppression to definitive cure with targeted antimicrobials – elegantly delineates the core pathophysiology: an aberrant, yet targeted, immune response to a persistent microbial trigger.

Furthermore, this case lends credence to the conceptual framework of a “tuberculous hypersensitivity spectrum.” Various cutaneous tuberculids, including PNT, erythema induratum, and lichen scrofulosorum, are increasingly regarded not as entirely discrete entities but as morphological variations of a shared underlying hypersensitivity state, differing primarily in the anatomical depth and caliber of the affected blood vessels. Reports documenting the simultaneous occurrence of PNT and lichen scrofulosorum lend support to this continuum model.^[30]^ Extending this paradigm, PD can be logically situated within this same spectrum as its articular counterpart. Therefore, our patient’s presentation may represent a more systemic and pronounced expression of this hypersensitivity diathesis, wherein the immune response concurrently targets both cutaneous and synovial tissues. This suggests that PNT and PD are not merely coincidental independent events, but rather are intrinsically linked manifestations on a broader pathophysiological continuum.

This clinical report yields substantial implications for medical practice, spanning diagnostic, therapeutic, and long-term management domains. Firstly, from a diagnostic perspective, this case accentuates the imperative of maintaining heightened clinical vigilance for TB-associated hypersensitivity syndromes in regions where the disease is endemic. The co-occurrence of refractory, necrotizing cutaneous lesions and an inflammatory, nonerosive polyarthritis should immediately raise suspicion for an underlying tuberculous etiology. A comprehensive diagnostic approach is paramount, integrating a meticulous clinical history (including potential exposure and constitutional symptoms), skin biopsy for histopathology and microbiological studies, relevant imaging (e.g., chest radiography, lymph node ultrasonography), and immunologic assays (such as tuberculin skin testing or interferon-gamma release assays). Critically, this case exemplifies that a protracted diagnostic delay, as witnessed in our patient, can lead to the chronicity of both dermatologic and articular manifestations, consequently escalating patient morbidity and the potential for irreversible tissue damage. The initial misdiagnosis and the transient partial response to methotrexate underscore the diagnostic pitfalls and the ease with which the true infectious trigger can be obscured.

Secondly, concerning therapeutic management, this case reaffirms ATT as the cornerstone and definitive intervention. The observed dramatic and parallel resolution of both skin and joint symptoms following the initiation of a 3-drug regimen comprising isoniazid, rifampicin, and ethambutol is consistent with established literature on these individual conditions.^[17,21]^ The role of immunomodulatory agents, such as methotrexate or corticosteroids, necessitates careful reevaluation. The initial, partial symptomatic relief afforded by methotrexate presents a clinical paradox; while providing temporary benefit, it inadvertently masked the underlying etiology, thereby prolonging the diagnostic odyssey and delaying the initiation of curative ATT. This sequence of events illustrates the potential for immunosuppressants to inadvertently facilitate disease progression in the context of an undiagnosed TB hypersensitivity syndrome.^[5]^ Therefore, in any suspected case of PD or PNT, ATT must be prioritized. Immunosuppressants should be employed with extreme caution, typically reserved as a short-term bridging strategy under stringent clinical supervision and concurrent with effective ATT.

Thirdly, long-term management strategies must account for the risk of recurrence or reactivation. Even after the successful completion of a full ATT course, patients should be counseled regarding the potential for relapse, particularly in the event of altered immune status (e.g., due to subsequent immunosuppressive therapies). Regular clinical follow-up is advisable to monitor for the reemergence of cutaneous or articular symptoms. Such vigilance is especially critical in patients like ours, who have experienced significant diagnostic delays, as their disease has already demonstrated a propensity for progression and chronicity. This underscores the importance of thorough patient education and sustained clinical awareness in individuals with a history of such profound TB hypersensitivity reactions.

As with any case report, our study is subject to inherent limitations that warrant acknowledgment to maintain scientific rigor. Primarily, the observations are derived from a single patient. While instructive, this design precludes the establishment of causality or determination of the prevalence of the PNT-PD association; our findings serve to generate hypotheses rather than to be generalized to a broader population. Secondly, the follow-up duration, albeit sufficient to demonstrate a robust initial therapeutic response, may be considered relatively short (e.g., 6–12 months). Longer-term surveillance is necessary to fully elucidate the durability of remission and the lifelong risk of sequelae or recurrence. Thirdly, our investigative scope was confined to clinical and routine pathological assessments. We did not perform advanced immunophenotyping, cytokine profiling, or human leukocyte antigen (HLA) genotyping, which could have offered deeper insights into the specific immune dysregulation and potential genetic predispositions underlying this dual presentation.^[6]^ Finally, a definitive pathological confirmation of the sterile nature of PD ideally necessitates synovial biopsy, which was not performed in our patient. While the compelling clinical presentation and unequivocal response to ATT are strongly indicative, the absence of direct synovial tissue analysis remains a minor diagnostic limitation.

## 9. Conclusion

This report provides definitive evidence of concurrent PNT and PD as a unified, systemic hypersensitivity response to MTB antigens. Our findings establish that synchronous cutaneous and articular manifestations reflect a disseminated Type IV hypersensitivity reaction, occurring within a unique immunologic context characterized by elevated IgE and an atypical Th1/T helper 2 (Th2) profile. While classical pulmonary TB typically exhibits Th1 dominance, our patient’s elevated IgE and clinical presentation align with literature documenting Th2-skewed responses in certain TB-related hypersensitivity syndromes or anergic states. The application of structured diagnostic criteria – including histopathological evidence of leukocytoclastic vasculitis and granulomatous inflammation, alongside Sharma and Pinto’s criteria for PD – enabled definitive classification and underscored the necessity of integrating immunologic and histologic data in atypical presentations.

These insights directly inform clinical practice. The parallel resolution of skin and joint symptoms with ATT confirms its centrality in management, while the transient response to MTX prior to ATT highlights the risks of empiric immunosuppression. In TB-endemic settings, clinicians should consider dual PNT-PD in patients with refractory cutaneous lesions and sterile arthritis, initiating ATT promptly to prevent chronicity. Future studies should prioritize immunologic biomarker discovery – such as cytokine signatures or HLA associations – to facilitate early diagnosis and personalized therapeutic strategies for systemic tuberculous hypersensitivity.

## 10. Patient perspective

As the patient, I recall the initial years of suffering as deeply frustrating. After being misdiagnosed with psoriatic arthritis, I was prescribed weekly methotrexate and topical corticosteroids. While these provided temporary relief for my rash and joint pain, new lesions kept emerging, and the symptoms would resurface despite ongoing treatment. This cycle left me feeling hopeless and confused – I couldn’t understand why the medications were not working long term. The recurrence 1 month before admission, with severe pustular lesions and arthralgia, only heightened my anxiety, especially when acitretin treatment failed to help. Looking back, the delay in diagnosis amplified my distress, as I endured years of uncertainty without answers.

The correct diagnosis of papulonecrotic tuberculid and Poncet’s disease was a turning point. Starting the 6-month antitubercular therapy with isoniazid, rifampicin, ethambutol, and intermittent celecoxib resulted in profound relief. Within weeks, my skin lesions began to heal, and the joint pain subsided significantly. By the end of treatment, I achieved near-complete remission. This experience filled me with gratitude – finally, a solution that worked. It underscores the importance of accurate diagnosis and has motivated me to share my story, hoping it helps others avoid similar delays.

## Acknowledgments

During the preparation of this manuscript, the authors used Kimi (Moonshot AI) for language editing and polishing of the text. All AI-generated content was subsequently reviewed, edited, and approved by the authors, who take full responsibility for the final manuscript.

## Author contributions

**Conceptualization:** Zhubiao Ye, Yujian Ye.

**Data curation:** Su Wang.

**Investigation:** Shuna Zhang, Zhubiao Ye.

**Resources:** Yujian Ye.

**Supervision:** Yujian Ye.

**Visualization:** Zhubiao Ye.

**Writing – original draft:** Zhubiao Ye.

**Writing – review & editing:** Li-Tian Ma, Yujian Ye.
